# Preharvest UVA-LED Enhancing Growth and Antioxidant Properties of Chinese Cabbage Microgreens: A Comparative Study of Single Versus Fractionated Irradiation Patterns

**DOI:** 10.3390/foods14234092

**Published:** 2025-11-28

**Authors:** Junxi Ai, Han Gao, Yamin Fan, Quan Yuan, Ran Wu, Ahmet Beyatli, Xiaoqiang Shi, Silvana Nicola, Shuihuan Guo, Hafiz A. R. Suleria, Lijuan Zhan

**Affiliations:** 1 College of Food Science and Technology, Henan Agricultural University (HAU), Wenhua Road 95, Zhengzhou 450002, China; aijunxixi0524@163.com (J.A.); gaoduo397@163.com (H.G.); fanyamin@126.com (Y.F.); yuanquan0818@163.com (Q.Y.); wuran1121@163.com (R.W.); 2 School of Agriculture, Food and Ecosystem Sciences, Faculty of Science, The University of Melbourne, Parkville, VIC 3010, Australia; ahmet.beyatli@sbu.edu.tr; 3 Medicinal and Aromatic Plants Program, Hamidiye Vocational School of Health Services, University of Health Sciences, 34668 Istanbul, Türkiye; 4 Zhengzhou Academy of Agricultural Science and Technology, Zhengzhou 450015, China; ashixiaoqiang123@163.com; 5 Department of Agricultural, Forest and Food Sciences, University of Turin, Largo Paolo Braccini, 2, 10095 Grugliasco, Italy; silvana.nicola@unito.it

**Keywords:** microgreens, UV-LED, irradiation, antioxidant quality, functional food, enzymes

## Abstract

Ultraviolet-A light-emitting diode (UVA-LED) irradiation is an emerging technology for biofortifying plants with enhanced nutraceuticals. This study firstly investigated effects of various doses (0-control, 16, 32, 48 J/cm^2^) on Chinese cabbage microgreens (CCM) quality, identifying 32 J/cm^2^ as the suitable dose for improving total antioxidant capacity (TAC) of CCM. Based on this dosage, the following two irradiation patterns were compared: single irradiation (SI, single pulse of 32 J/cm^2^) and fractionated irradiation (FI; four pulses of 8 J/cm^2^ each). Both FI and SI significantly enhanced CCM quality, though through distinct mechanisms. FI effectively promoted accumulation of biomass and vitamin C, with increases by 9.25% and 13.20%, respectively. Meanwhile, SI markedly enhanced 20.90% higher TAC than FI. This was achieved by elevating enzymatic (7.71% superoxide dismutase-SOD, 9.03% peroxidase-POD, 40% catalase-CAT, and 52.17% ascorbate peroxidase-APX) and non-enzymatic (18.89% total phenolics-TPC, 10.04% total flavonoids-TF, and 18.99% carotenoids) antioxidants. Additionally, both FI and SI significantly reduced the nitrate content. To our knowledge, this is the first study to demonstrate the effect of UVA-LED irradiation pattern on microgreens quality. These findings provide basic information for UVA-LED application in indoor agriculture and the food industry, emphasizing the importance of strategically selecting irradiation patterns to achieve specific production goals.

## 1. Introduction

Microgreens are edible seedings of various plants, typically harvested 7–20 days after germination, once they reach a height of 2 to 7 cm, depending on species. At this stage, the plants generally exhibit the first pair of true leaves [1]. Microgreens are rich in pigments, minerals, vitamins, carotenoids, phenolic compounds, glucosinolates, and other bioactive components. In fact, the young plants like microgreens contain almost 20-fold more bioactive compound content than their mature counterparts [2]. These phytochemical compounds exhibit various health-promoting and disease-preventing properties arising from their antioxidant properties, such as fighting inflammation, cancer prevention, and improved heart health and digestion. Given these benefits, microgreens are considered as an emerging functional food [3]. The global microgreens market attained a valuation of $1.77 billion in 2024 and is projected to reach $3.27 billion by 2033, growing at an annual growth rate at 6.97% from 2025 to 2033 [4]. This growth is largely driven by the growing consumer awareness of the nutritional benefits and a rising preference for fresh, locally sourced produce [4].

About 80–100 crop varieties can be cultivated as microgreens [5]. The most commonly growing species come from twelve plant families [1], with varieties from the Brassicaceae family capturing a significant share of the market. This dominance is attributed to their robust flavor and richness of phytochemicals such as phenolic compounds, vitamins, glucosinolates, isothiocyanates, and indoles [4,6]. Among the Brassicaceae, species such as cabbage, broccoli, and radish are the most extensively studied due to their exceptional diversity of bioactive compounds. These species are also the most commercially widespread microgreens, favored for their premium quality, consumer acceptability, and the broad availability of seeds [6].

Microgreens are suitable for both home cultivation and large-scale commercial production. They require minimal space, consume less water, and generally do not require fertilizers or pest and weed control. With these advantages, microgreens production is particularly well-suited for controlled environment agriculture (CEA) such as indoor vertical farming (VF). However, one of the limitations of indoor cultivation system is the lack of ultraviolet (UV) radiation, which adversely affects the nutritional quality of plants, mostly reducing the level of phenolic compounds [7]. This challenge can potentially be addressed through the application of UV light emitting diode (UV-LED) technology.

In the past few years, new generations of UV-LED irradiation have emerged as a promising and innovative alternative to biofortify plant with improved nutraceuticals [2]. Compared to traditional UV lamps, UV-LEDs have the advantages of higher energy efficiency, lower heat generation, small size, long lifespan, and being environmentally friendly (free of ozone, mercury, and breakable glass) [8]. Notably, their narrow bandwidth enables precise selection of peak wavelengths allowing researchers to explore the specific effects of individual types of UV radiation while eliminating cross-interference among different UV spectra. Typically, UV irradiation comprises three portions, UVA (320–400 nm), UVB (280–320 nm), and UVC (200–280 nm). Among these, UVA is the predominant form of natural UV radiation reaching the plants, as the ozone layer absorbs most UVB and entirely UVC [9]. Moreover, UVA is less destructive to plants and safer to handle due to its longer wavelength and lower energy, making it especially manageable and suitable for the application in CEA facilities [10]. In terms of these advantages, UVA-LED is applied as a preharvest treatment to stimulate the growth and enhance the level of antioxidant compounds in leafy vegetables. Current studies have mainly focused on effects of UVA wavelengths peaks [10], irradiation durations [11], light intensity [12,13], as well as combination of UVA with other UV spectrum [14] on growth and quality of leafy greens. However, no studies have explored plant responses to different irradiation patterns of UVA-LEDs across the literature. Earlier studies have shown that, compared to single irradiation, fractionated irradiation using low dose of gamma ray (*γ*-ray) could promote plant growth and height, as well as increase fresh weight and dry matter content [15,16]. However, Silva et al. [17] recently demonstrated that a single application of UVC at hermetic dose (0.3 kJ/m^2^) stimulated growth and productivity of red mustard microgreens, whereas fractionated irradiation (three pulses with 0.3 kJ/m^2^ each) showed no significant effects compared to controls. These conflicting findings indicate that plant growth and quality are not only affected by species and parameters of irradiation per se, but also by how the irradiation is applied. Nevertheless, to date, and to the best of our knowledge, no study has compared the effect of different UVA-LED irradiation patterns on growth and antioxidant quality of microgreens.

To address this gap, this study aimed to investigate the influence of preharvest UVA-LED irradiation patterns on growth and antioxidant property of Chinese cabbage microgreen (CCM) by carrying out two separate trials. The first experiment focused on identifying the suitable dose by comparing the impact of different doses (0-control, 16, 32, and 48 J/cm^2^) on growth and total antioxidant capacity (TAC) in CCM. Based on the suitable 32 J/cm^2^ dose identified, the second trial compared the impact of two distinct irradiation patterns: single irradiation (SI, a single pulse of 32 J/cm^2^ at 20 days after sowing) and fractionated irradiation (FI, four pulses of 8 J/cm^2^ each at 14, 16, 18, and 20 days after sowing)-on growth and antioxidant property of CCM. The findings not only provide new insights into how microgreens respond to different stimulation patterns of UVA-LED but propose a practical and innovative strategy to enhance microgreens production in CEA system using UVA-LED technology.

## 2. Material and Methods

### 2.1. Microgreen Cultivation and Treatments

#### 2.1.1. Growth Condition

**Figure 1:**
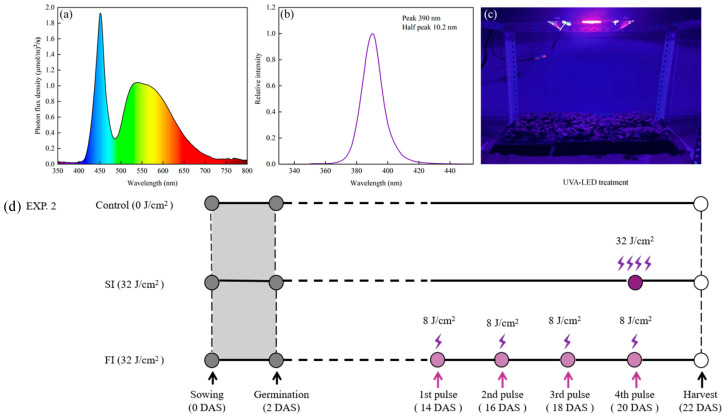
The light condition and treatments. (**a**), White light spectrum for growth; (**b**), UVA-LED wavelength peak; (**c**), UVA-LED irradiation treatment; (**d**), UVA-LED irradiation patterns treatment, SI—single irradiation, FI—fractionated irradiation; DAS—days after sowing.

Chinese cabbage (*Brassica rapa* subsp. *pekinensis*) seeds were obtained from Zhengzhou Academy of Agricultural Science and Technology and manually sown in a polystyrene 60-cell tray (24 cm × 16 cm) filled with peat-based horticultural medium. Between 10 and 15 seeds were sown per cell. After germination under dark condition (2 days after sowing), the trays were transferred into a customized climate chamber, where the LED white light (Figure 1a) was set 12 h photoperiod (8 am to 8 pm) and light intensity of approx. 90 μmol m^−2^ s^−1^ at plants level. Thinning was performed on 7 days after sowing, retaining only 6–7 plants per cell. During plants’ growth period, the temperature in chamber was set approx. 24 ± 0.5 °C during the daytime and 18 ± 0.5 °C during the night, with relative humidity ranging from 60% to 72%. Plants were irrigated as required with tap water only.

#### 2.1.2. UVA-LED Irradiation Dose Selection

In the first experiment (Figure S1), plants were subjected to preharvest UVA-LED irradiation at 14, 16, 18, and 20 days after sowing (DAS) using a customized UVA-LED system with a peak wavelength of 390 nm (Figure 1b,c). The distance from the UVA-LED panel to the leaf is 37 cm. The irradiation intensity at the plant canopy level was measured at 12 mW/cm^2^. The corresponding dose of 0-control, 16, 32, 48 J/cm^2^ was obtained by multiplying irradiation intensity by irradiation time (Table S1). The irradiation treatments were conducted within the same room of cultivation chamber. During UVA-LED application, plant trays were manually transferred to the irradiation apparatus, which was positioned separately from the cultivation system (Figure 1c). Given the minimal transfer distance, no specific protective measures were taken during plant relocation. After irradiation, the plants were immediately returned into the climate chamber to continue cultivation. The background white light illumination was maintained continuously throughout irradiation phases. On the harvest day (22 DAS), microgreens were cut at the substrate level using sharp scissors. Growth performance parameters including plant height, biomass, chlorophyll, dry matter content, and total antioxidant capacity (TAC) were analyzed.

#### 2.1.3. UVA-LED Irradiation Patterns Comparison

Based on the suitable irradiation dose (32 J/cm^2^) identified in Experiment 1, the second experiment (Figure 1d) was carried out to further compare the effects of irradiation patterns on growth and antioxidant property of CCM. In this case, the following three irradiation patterns were applied: (1) control, no UVA-LED irradiation, (2) single irradiation (SI): a single pulse of 32 J/cm^2^ dose at 20 DAS, and (3) fractionated irradiation (FI): four pulses of 8 J/cm^2^ dose each at 14, 16, 18, and 20 DAS (Figure 1d; Table S2). The irradiation treatments and harvest procedures followed those described in Experiment 1. On the harvest day (22 DAS), plant growth performance and growth parameters including biomass, dry matter (DM), chlorophyll-(*Chl*) were monitored. Antioxidant enzyme activity (superoxide dismutase—SOD, catalase—CAT, peroxidase—POD, and ascorbate peroxidase—APX), antioxidant compounds (carotenoids, ascorbic acid—AA, total phenolic compounds—TPC, total flavonoid content—TFC), and total antioxidant capacity (TAC) were assayed. Additionally, the content of malondialdehyde (MDA) and nitrate were also analyzed.

### 2.2. Growth Parameters Assay

#### 2.2.1. Growth Morphology and Agronomy Traits

The plants’ growth performance was documented through photography on the harvest day. Plant morphology was monitored by scanning samples using a Canon scanner. Plant height was measured using a ruler as the vertical distance (cm) from the base of the plant (soil surface) to the apex of the main stem, excluding extended leaves. Biomass was calculated as the ratio of the aboveground fresh weight (kg) of microgreens in each replicate to the corresponding area (m^2^) and expressed as kg/m^2^. DM content was determined by drying 5 g of fresh leaves in an oven at 65 °C until a constant weight was achieved.

#### 2.2.2. Chlorophyll and Carotenoid Assay

Chlorophyll and carotenoids content were measured following our previous method [18] with slight modification. Briefly, 1 g of fresh tissue was ground in 5 mL of 95% ethanol, and the homogenate was extracted overnight in refrigerator at 4 °C in darkness until tissue became completely colorless. The extract was then centrifuged at 8000 r/min at 4 °C for 10 min. The supernatant was analyzed spectrophotometrically for *Chl a*, *Chl b*, and carotenoid content at wavelengths of 665, 649, and 470 nm, respectively.

### 2.3. Antioxidants and Total Antioxidant Capacity Assay

#### 2.3.1. Total Phenolic Compounds and Total Flavonoid Content Assay

TPC was assayed using the Folin–Ciocalteu procedure with slight modification based on our protocol [18]. An amount of 1 g of fresh tissue was homogenized in 3.5 mL of pre-cooled 95% methanol. The homogenate was incubated at 4 °C for 2 h, with shaking every 1 h. The extract was then centrifuged at 8000 r/min for 20 min at 4 °C. An amount of 0.1 mL aliquot of the supernatant was mixed with 0.5 mL of Folin–Ciocalteu reagent and 0.9 mL of 7.0% (*w*/*v*) NaCO_3_ solution. The mixture was incubated in a 20 °C water bath for 30 min and the absorbance measurement at 760 nm. A gallic acid standard curve was established to quantify TPC.

The total flavonoid content (TFC) was determined using the aluminum chloride colorimetric method, as described by Chang et al. [19] with modification. An amount of 0.1 mL of the methanol extract was mixed with 1 mL of 95% methanol, and 0.1 mL of 10% aluminum chloride (AlCl_3_) solution, and 0.1 mL 1 mM potassium acetate (CH_3_COOK). The mixture was thoroughly stirred and incubated for 30 min at 25 °C before the absorbance was measured at 420 nm. A calibration curve was established using rutin standard solutions ranging from 30 to 100 µg/mL, and the results were expressed as milligrams of rutin equivalents per kilogram of fresh weight (mg rutin/kg FW).

#### 2.3.2. Total Ascorbic Acid and Ascorbic Acid

Total ascorbic acid (TAA) and ascorbic acid (AA) contents were determined spectrophotometrically following the method of Kampfenkel et al. [20], with slight modifications. Briefly, 1 g of fresh microgreens was ground in 3.5 mL of 6% ice-cold trichloroacetic acid (TCA) solution. The homogenate was centrifuged at 8000 rpm for 20 min at 4 °C, and the resulting supernatant was immediately collected for the analysis of TAA and AA. The content of dehydroascorbic acid (DHA) was calculated as the difference between TAA and AA. The results were expressed as milligrams per kilogram of fresh weight (mg/kg FW), based on calibration curves constructed using standard solution of *L*-ascorbic acid (25–250 mg/L).

#### 2.3.3. Total Antioxidant Capacity Assay

In this experiment, two methods were used to determine the total TAC of CCM, namely ABTS (2,2-azinobis (3-ethyl-benzothiazoline-6-sulfonic acid) [21] and DPPH (2,2-diphenyl-1-picrylhydrazyl) [22]. The extraction procedure followed the method described in Section 2.3.1. For the ABTS assay, fresh ABTS^●+^ working solution was prepared by mixing 7 mM ABTS^●+^ and 2.45 mM potassium persulfate in equal volume, followed by 12 h incubation in darkness. For each assay, 0.1 mL of the methanol extract was mixed with 1.9 mL of the ABTS^●+^ working solution. After incubating at 25 °C for 6 min, absorbance was read at 734 nm. Results were expressed as micromoles of Trolox equivalents per kilogram fresh weight tissue (µmol Trolox/kg FW) based on a standard curve constructed with Trolox solutions (20 to 160 µM).

For the DPPH assay, a working solution was freshly prepared by diluting the DPPH stock with methanol to an initial absorbance of 1.1 ± 0.02 at 515 nm. For the assay, 0.1 mL extract was mixed with 1.90 mL diluted solution. The mixture was incubated at 37 °C for 30 min in the dark, after which the absorbance was recorded at 515 nm. The results were expressed as micromoles of Trolox equivalents per kilogram fresh weight (μmol Trolox/kg FW).

### 2.4. Antioxidant Enzyme Activities and MDA Content Assay

#### 2.4.1. Antioxidant Enzyme Activities Assays

Enzyme extraction was conducted according to our previously method [18]. Briefly, 0.5 g fresh plant was grounded in 4 mL of pre-cooled 50 mM phosphate-buffer solution (PBS, pH 7.8) containing 2% polyvinylpyrrolidone (PVP) and 0.2 mM ethylenediaminetetraacetic acid (EDTA). The homogenate was centrifuged at 8000 r/min for 20 min at 4 °C. The supernatant was then collected as a crude enzyme extract for the determination of SOD, CAT, POD, and APX activities.

SOD activity was determined by measuring the enzyme’s capacity to inhibit photochemical reduction in nitro blue tetrazolium (NBT) at 560 nm [23]. In this assay, a 50 μL of enzyme extract was added to 3 mL of NBT reaction solution that contained 0.75 mM NBT, 130 mM L-methionine, and 0.1 mM EDTA in 50 mM PBS (pH 7.8). The reaction was initiated by adding 1 mL 20 μmol/L riboflavin, and mixture was immediately exposed to uniform light condition (90 µmol·m^−2^ s^−1^) for 30 min at 25 °C. Absorbance was measured at 560 nm. One unit (U) of SOD activity was defined as the amount of enzyme that inhibits the reduction in NBT by 50%. The specific SOD activity was expressed as U per gram fresh weight (U/g FW).

CAT activity was determined based on method of Cakmak and Marschner et al. [24] with slight modification. Briefly, 0.1 mL of crude extract was mixed with 1.7 mL of 25 mM PBS (pH 7.0, containing 0.1 mM EDTA). The reaction was initiated by adding 0.2 mL of 100 mM H_2_O_2_ solution. The decrease in absorbance was recorded at 240 nm. One unit (U) of CAT activity was defined as the amount of enzyme required to decrease the absorbance by 0.01 per minute, and results were expressed as U per gram fresh weight (U/g FW).

POD activity was determined according to our previous description with modification [18]. The POD reaction mixture consisted of 1.7 mL of 25 mM PBS, 100 μL of 1% guaiacol, 100 μL of 20 mM H_2_O_2_, and 0.1 mL of crude enzyme extract. POD activity was measured as an increase in absorbance at 470 nm and expressed as a unit per gram of fresh weight (U/g FW).

APX was determined according to the method of Shibaeva et al. [25] with slight modifications. For each assay, 0.1 mL crude extract was mixed into 1.7 mL of 25 mM PBS. Following that, 0.1 mL of 5 mM ascorbic acid was added. The reaction was initiated by adding 0.1 mL of 20 mM H_2_O_2_, and the decrease in absorbance was recorded at 290 nm. One unit (U) of APX activity was defined as the amount of enzyme required to decrease the absorbance by 0.01 within 30 s. Enzyme activity was expressed as U per gram fresh weight (U/g FW).

#### 2.4.2. MDA Content Assay

MDA assay was assayed referring to Gao et al. [26] with slightly modifications. Fresh plant tissue (0.5 g) was homogenized in 5.0 mL of 10% TCA solution. The homogenate was centrifuged at 8000 r/min for 20 min, and the supernatant was collected. Then, 2 mL of supernatant was mixed with 2 mL 0.67% (*w*/*v*) thiobarbituric acid (TBA) solution. The mixture was incubated in a boiling water bath at 100 °C for 20 min. After cooling in room temperature, the mixture was centrifuged again. The absorbance of the supernatant was measured at 450 nm, 532 nm, and 600 nm, respectively. MDA content was expressed as micromoles per kg fresh weight (µmol/kg FW).

### 2.5. Nitrate Content Assay

Nitrate content was determined according to ultraviolet spectrophotometric method in Agricultural Industry Standard of China [27] with slight modification. Briefly, 0.5 g of fresh plant tissue was homogenized with 20 mL of deionized water. To the homogenate, 1 mL of ammonia buffer (pH 9.6) and 0.4 g of activated carbon were added to eliminate pigment interference. The mixture was shaken for 30 min, followed by the addition of 0.4 mL of 15% (*w*/*v*) potassium ferrocyanide (K_4_Fe(CN)_6_·3H_2_O) and 0.4 mL of 30% (*w*/*v*) zinc sulfate (ZnSO_4_) solutions. The final volume was adjusted to 50 mL with deionized water, thoroughly mixed, and allowed to stand for 5 min. The suspension was subsequently filtered through quantitative filter paper and the resulting filtrate was collected for analysis. Absorbance was measured at 219 nm. Nitrate concentration was calculated based on a standard curve prepared from freshly dissolved potassium nitrate (KNO_3_) over the range of 2–12 mg/L. Results were expressed as milligrams per kg of fresh weight (mg/kg FW).

Unless stated otherwise, all homogenization procedures were carried out using a high throughput tissue grinder (SCENTZ-48, Ningbo Xinzhi Biotechnology Co., Ltd., Ningbo, China). Centrifugation was performed using the refrigerated high-speed centrifuge (3K30, Sigma Laborzentrifugen GmbH, Osterode, Germany); All spectrophotometric measurements were conducted with UV-visible spectrophotometer (JH754PC, Shanghai Jinghua Technology Instrument Co., Ltd., Shanghai, China). The reagents of Folin–Ciocalteu reagent, ABTS, DPPH, and guaiacol were purchased from Sangon Biotech (Shanghai, China) Co., Ltd. The standard substances of Trolox, garlic acid, and rutin were obtained from Weiye Metrology (Weiye Metrology and Technology Research Group Co., Ltd., Xinyang, China)

### 2.6. Statistical Analysis

Statistical analysis was conducted using SPSS (Version 27.0). One-way analysis of variance (ANOVA) followed by the Duncan test (*p* ≤ 0.05) was applied to determine significant differences among treatment means. Data were presented as boxplots that were generated using Origin (version 2024). Pearson correlation analysis was performed to assess relationships between variables, with results expressed as correlation coefficients (R) and corresponding *p* values. Multivariate principal component analysis (PCA) was performed using SPSS (Version 27.0) software, and the PCA results (Biplot, loading plot) were generated using Origin (Version 2024). Heat maps were generated using Chiplot (https://www.chiplot.online/heatmap.html) (accessed on 17 July 2025).

## 3. Results

### 3.1. Selection of Irradiation Dose

**Figure 2:**
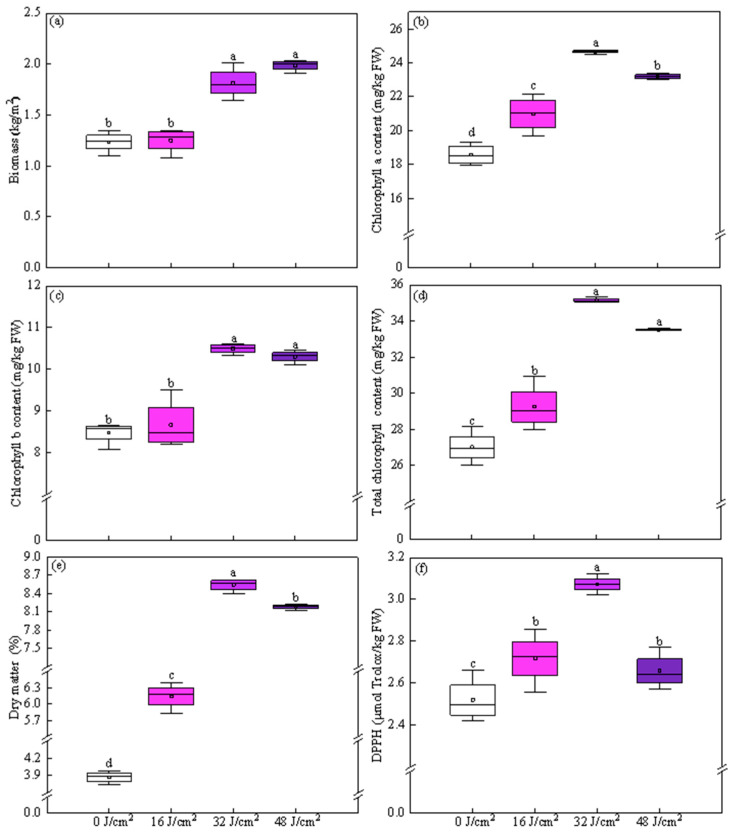
Effect of UVA-LED irradiation doses on growth traits of Chinese cabbage microgreen. ((**a**), Biomass; (**b**), Chlorophyll a; (**c**), Chlorophyll b; (**d**), Total chlorophyll; (**e**), Dry matter; (**f**), DPPH-2,2-diphenyl-1-picrylhydrazyl). Small square within each box represents the mean value. Different lowercase letters indicate significant differences among irradiation doses (*p* < 0.05).

Pre-harvest application of UVA-LED irradiation significantly influenced the morphological characteristics and growth of CCM, with effects being highly dose-dependent (Figure 2 and Figure S2). The plants treated with UVA-LED exhibited deeper green coloration than those from the control (Figure S2). These visual improvements further aligned to the significantly higher chlorophyll levels in UVA-LED treated plants. As shown in Figure 2a, the biomass (up-ground fresh weight) exhibited a significant dose-dependent response to UVA-LED irradiation. Treatments of 32 and 48 J/cm^2^ significantly increased biomass by 47.16% and 61.02%, respectively, compared to the control (*p* < 0.05); however, no significant difference was observed between these two doses. In contrast, the treatment of 16 J/cm^2^ dose did not exhibit significant influence on biomass compared to the control (Figure 2a).

All UVA-LED treatments significantly increased *Chl a* content compared to the control, demonstrating increases of 11.46%, 33.47%, and 25.74% at doses of 16, 32, and 48 J/cm^2^, respectively (Figure 2b). Among these, the 32 J/cm^2^ dose resulted in the highest *Chl a* content, which was significantly higher by 19.74% and 6.15% compared to the 16 and 48 J/cm^2^ treatments, respectively. Differing from *Chl a*, only treatments of 32 and 48 J/cm^2^ significantly increased *Chl b* content, which increased by 22.61% and 20.37%, respectively, compared to the control (Figure 2c). No significant difference was observed between treatments of 32 and 48 J/cm^2^. Total *Chl* content increased progressively with increased irradiation dose in the range of 16 to 32 J/cm^2^, then reached a plateau at 48 J/cm^2^, showing no further significant increase compared to the 32 J/cm^2^ dose. Specifically, treatments of 16, 32, and 48 J/cm^2^ doses resulted in increases by 8.25%, 30.04%, and 24.05% in total *Chl* content compared to control, respectively. Total *Chl* content in plants treated by 32 and 48 J/cm^2^ doses were statistically the same, and both were significantly higher than those in plants treated by 16 J/cm^2^ dose and control (Figure 2d).

Regarding dry matter (DM) accumulation, all UVA-LED irradiation doses applied significantly promoted DM content in CCM (Figure 2e). The DM content of plants treated the 16, 32, and 48 J/cm^2^ was 1.59-, 2.21-, and 2.11-fold that of the control, respectively. Among these, the 32 J/cm^2^ dose treatment was the most effective, resulting in 39.02% and 4.37% higher DM than the treatments of 16 and 48 J/cm^2^, respectively. The plant height was not significantly influenced by any of UVA-LED treatments, maintaining an average value of 4.74 cm (Figure S2c).

As expected, TAC values, assayed using DPPH method, were induced to increase by all UVA-LED treatments, showing increases by 7.90% for 16 J/cm^2^, 21.95% for 32 J/cm^2^, and 5.52% for 48 J/cm^2^ treatments, respectively, compared to control (Figure 2f). Apparently, 32 J/cm^2^ was the most efficient in enhancing TAC values, yielding increases of 13.02% and 15.58% compared to the 16 J/cm^2^ and 48 J/cm^2^ treatment, respectively; the latter two treatments showed no significant difference in TAC values.

Conclusively, all UVA-LED doses applied in range of 16–48 J/cm^2^ showed beneficial effects on enhancing the growth and antioxidant quality of CCM. Nevertheless, the extent varied across the treatments and was highly dose dependent. Among them, 32 J/cm^2^ was identified as the most effective, as it significantly resulted in higher biomass, chlorophyll content, DM, as well as TAC levels than 16 J/cm^2^. Meanwhile, the 32 J/cm^2^ treatment significantly improved the levels of *Chl a*, DM, and TAC compared to 48 J/cm^2^. Based on these facts, 32 J/cm^2^ was selected as suitable irradiation dose and subsequently applied in the second experiment to evaluate its effects, under different irradiation patterns, on growth and antioxidant property of CCM.

### 3.2. Effect of Irradiation Patterns on Growth Traits

**Figure 3:**
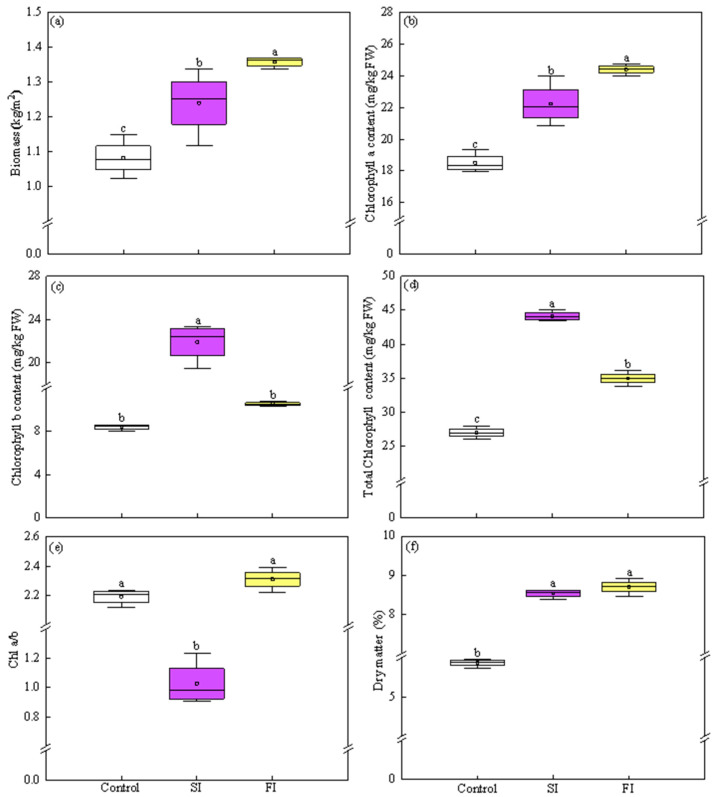
Effect of UVA-LED irradiation patterns on growth traits of Chinese cabbage microgreen. ((**a**), Biomass; (**b**), Chlorophyll a; (**c**), Chlorophyll b; (**d**), Total chlorophyll; (**e**), *Chl a*/*b*−Chlorophyll a/b; (**f**), Dry matter). Small square within each box represents the mean value. Different lowercase letters indicate significant differences among irradiation patterns (*p* < 0.05).

Based on the results from the first experiment, the second experiment investigated the effects of the following two irradiation patterns: SI (one pulse, 32 J/cm^2^) and FI (four pulses, 8 J/cm^2^ each) on the growth and antioxidant quality of CCM (Figure 1d). The results showed that both FI and SI treatments significantly stimulated CCM growth, resulting in overall vigorous growth performance, compared to the control (Figure S3), indicating UVA-LED irradiation at 32 J/cm^2^ dose was effective in promoting CCM development. Specifically, both FI and SI treatments elevated biomass by 25.51% and 14.60%, respectively, compared to the control (*p* < 0.05). Moreover, FI resulted in significant 9.52% increases in biomass when compared to SI (Figure 3a).

In terms of chlorophyll content, plants treated with FI and SI exhibited increases of 31.83% and 20.12% in *Chl a*, 25.07% and 1.59-fold in *Chl b*, as well as 29.71% and 63.69% in total *Chl*, respectively, compared to those in the control plants (Figure 3b–d). Notably, FI-treated plants showed more *Chl a* (+9.75%), but lower *Chl b* (−51.74%) and total *Chl* (−20.76%) content compared to those treated with SI. Such elevation in total *Chl* content in SI-treated plant primarily resulted from the markedly greater increase in *Chl b* level (Figure 3b–d). As shown in Figure 3e, SI treatment resulted in a significant decrease in *Chl a*/*b* ratio of 53.66% compared to the control, whereas the FI treatment did not significantly affect *Chl a*/*b* ratio. Consequently, the *Chl a*/*b* ratio in SI-treated plant was 56.03% lower than that observed in FI.

Both FI and SI treatments significantly increased DM content by 48.28% and 45.47%, respectively, compared to the control (Figure 3f). No significant difference was observed between the two treatments.

### 3.3. Effect of Irradiation Pattern on Antioxidants and TAC

#### 3.3.1. TAA, AA, and DHA Content

**Figure 4:**
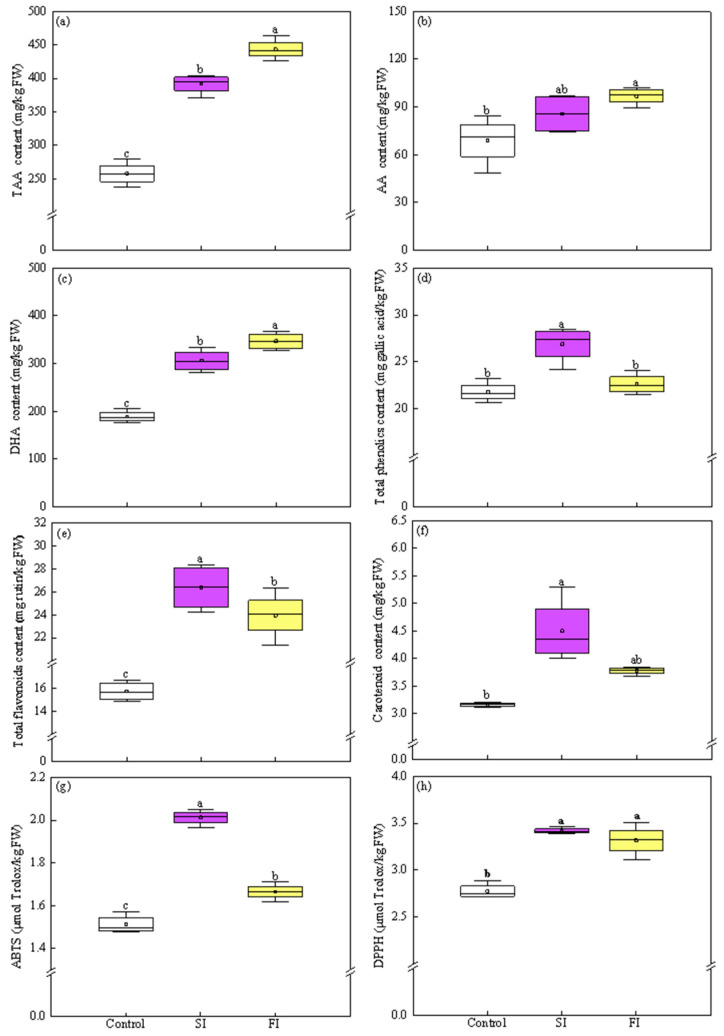
Effect of UVA-LED irradiation patterns on antioxidants and total antioxidant capacity (TAC) of Chinese cabbage microgreen ((**a**), TAA—total ascorbic acid; (**b**), AA—ascorbic acid; (**c**), DHA—dehydroascorbic acid; (**d**), TPC—total phenolics content; (**e**), TFC—total flavonoids content; (**f**), Carotenoid content; (**g**), ABTS—2,2′-azino-bis (3-ethylbenzothiazoline-6-sulfonic acid); (**h**), DPPH—2,2-diphenyl-1-picrylhydrazyl). Small square within each box represents the mean value. Different lowercase letters indicate significant differences among irradiation patterns (*p* < 0.05).

Both FI and SI treatments demonstrated the potential benefits in inducing accumulation of TAA, AA, and DHA in CCM (Figure 4a–c). In detail, FI and SI significantly enhanced the levels of TAA by 71.97% and 51.91%, AA by 40.44% and 24.25%, and DHA by 83.51% and 61.94%, respectively, compared to control. When compared to SI, FI was more effective in promoting the accumulation of TASA and DHA, increases of 13.20% and 13.32%, respectively (Figure 4a,c). However, no statistically significant difference in AA content was observed between FI and SI treatments. In spite of this, FI resulted in a 13.03% higher AA level than SI (Figure 4b).

#### 3.3.2. TPC, TF, and Carotenoids Content

The irradiation patterns of UVA-LED had differential effects on TPC. SI treatment significantly increased TPC by 23.08% compared to the control (*p* < 0.05), whereas FI did not lead to a statistically significant change in TPC (Figure 4d). As a result, SI-treated plants accumulated 18.89% more TPC than those treated with FI (Figure 4d). Different from the trend of TPC, TF content was significantly affected by both FI and SI, with increases of 52.52% and 67.84%, respectively, compared to the control. Despite both treatments being effective, SI was more favorable for stimulating TF accumulation, leading to a significantly higher TF content (+10.04%) than FI treatment (Figure 4e). The carotenoids content was significantly affected only by SI treatments, which induced a 42.24% increase compared to the control (Figure 4f). FI treatment did not result in the statistically significant change in carotenoids content compared to the control. Consequently, SI-treated plants showed 18.99% higher carotenoid content than FI-treated plants, though this difference was not statistically significant due to the high standard deviations (Figure 4f).

#### 3.3.3. TAC Values

The TAC values, assayed using both ABTS and DPPH method, were significantly enhanced by both FI and SI treatments, but to different extents (Figure 4g,h). In the ABTS assays, both SI and FI treatments significantly increased TAC values by 33.22% and 10.19%, respectively, compared to the control. Notably, SI was more effective in stimulating TAC level, resulting in 20.90% higher than FI (Figure 4g). In DPPH assay, both FI and SI significantly increased TAC values by 26.20% and 23.17%, respectively, compared to the control. However, the two treatments did not exhibit statistical difference (Figure 4h).

### 3.4. Effect of Irradiation Pattern on Antioxidant Enzymes and MDA

**Figure 5:**
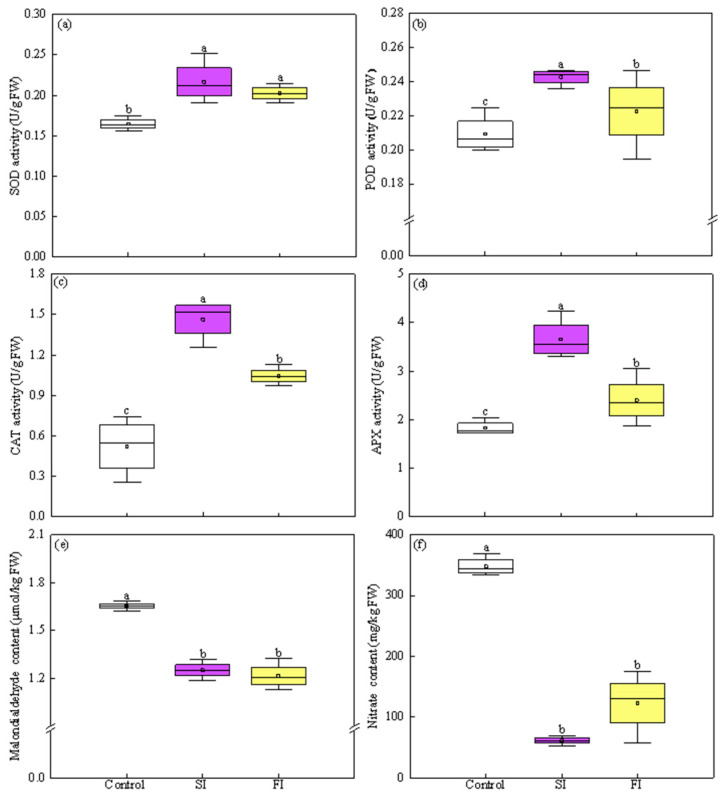
Effect of UVA-LED irradiation pattern on antioxidant enzyme activities, MDA, and nitrate content of Chinese cabbage microgreen ((**a**), SOD—superoxide dismutase; (**b**), POD—peroxidase; (**c**), CAT—catalase; (**d**), APX—ascorbate peroxidase; (**e**), MDA—Malonaldehyde; (**f**), Nitrate). Small square within each box represents the mean value. Different lowercase letters indicate significant differences among irradiation patterns (*p* < 0.05).

As shown in Figure 5a–d, preharvest application of UVA-LED with both FI and SI patterns significantly activated the activities of antioxidant enzymes. Specifically, FI and SI increased by 22.18% and 31.59% in SOD activities, respectively, compared to the control. There was no statistical difference in SOD activity between the two treatments, but SI treatment resulted in higher (+7.71%) SOD value than FI (Figure 5a). Similarly, FI and SI significantly increased activities of POD by 6.36% and 15.97%, CAT by 100% and 180%, and APX by 31.43% and 100%, respectively, compared to the control (Figure 5b–d). Apparently, SI was more effective than FI in enhancing these enzyme activities, resulting in significantly higher levels of POD (+9.03%), CAT (+40%), and APX (+52.17%) than FI.

MDA content, a key indicator of membrane lipid peroxidation, was markedly reduced by both FI and SI treatments, showing a decrease of 26.54% and 24.37%, respectively, compared to control. No significant difference in the MDA content was observed between treatments of FI and SI (Figure 5e).

### 3.5. Effect of Irradiation Pattern on Nitrate Content

The highest nitrate content in CCM was detected in control plants, being 347.77 mg/kg FW. UVA-LED treatments significantly reduced nitrate accumulation in CCM regardless of irradiation patterns (Figure 5f).

## 4. Discussion

### 4.1. UVA-LED Promotes the Growth and Total Antioxidant Capacity of CCM, but Strongly Dose-Dependent

To date, plant responses to UVA have been poorly understood, particularly when compared to other UV lights such as UVB [9]. The existing literature contains contradictory information, with studies often lacking detailed information on UVA spectrum and dose [9,18]. These inconsistencies can partly be attributed to limitations in earlier experimental designs, which lacked access to precise and tunable LEDs technology [12]. Indeed, only in recent years, the advances in LED technology have enabled precise control of both spectrum and intensity. Theoretically, this development provides a possibility for growers and researchers to explore plant responses to separate UVA irradiation without interference of other UV light spectrum. Yet, at present, a comprehensive understanding of UVA-induced changes in plant physiology and biochemistry is still lacking [9]. According to the current literature, the perception and response of plants to UVA radiation are species-specific and highly dependent on the spectral range and radiation dose, varying from stimulation to inhibition [5,9]. In the current study, UVA-LED irradiation within the range of 16–48 J/cm^2^ showed beneficial effect on CCM production, not only stimulating growth performance, but also enhancing total antioxidant property (Figure 2 and Figure S2). However, such desirable influence was highly dose-dependent, that is, a dose of 32 J/cm^2^ was the most effective, resulting in significantly higher levels of chlorophyll a, DM, and TAC compared to 16 and 48 J/cm^2^ treatments (Figure 2b,e,f), highlighting the importance of carefully considering the appropriate irradiation dose when applying UVA-LED technology in CEA and the food industry.

As shown in Figure 2, plant growth traits (biomass, chlorophyll, and DM) and antioxidant quality improved progressively from 16 to 32 J/cm^2^ compared to the control. However, further increasing the dose to 48 J/cm^2^ did not yield additional benefits. In fact, 48 J/cm^2^ dose irradiation led to noticeable declines in levels of *Chl a*, DM, and TAC (Figure 2b,e,f), as well as a plateau in levels of biomass and total *Chl* compared to 32 J/cm^2^ (Figure 2a,d). This finding suggested that the response of CCM to UVA-LED irradiation was a non-linear dose-response relationship with the increased UVA doses, implying both insufficient and excessive UVA doses can be detrimental. This observation was not consistent with the common assumption that 1% increment in light results in 0.5–1% yield increase for most crops [28]. This inconsistency is likely due to the extremely low photosynthetic quantum yield of UVA-LED light compared to visible radiation [29]. In line with our findings, Chen et al. [12] found that biomass and leaf area of lettuce did not increase linearly under UVA irradiation (365 nm) ranging from 10 to 30 µmol m^−2^ s^−1^. In contrast, signs of photoinhibition were observed at the high intensity of 30 μmol m^−2^ s^−1^, suggesting a saturating light response [12]. In agreement to our findings, He et al. [30] observed dose-dependent responses of Chinese kale to UVA irradiation. Their study illustrated that a medium dose of UVA-10 (380 nm, 10 W/m^2^) was optimal for producing high-quality Chinese kale leaves. Conversely, a lower dose of UVA-5 reduced photosynthate contents, while a higher dose of UVA-15 inhibited biomass accumulation and caused photosynthetic damage. In our study, no reduced biomass or visible photodamage symptoms on plant leaves were observed under 48 J/cm^2^ irradiation (Figure S2).

### 4.2. Both FI and SI Positively Affect Growth and Antioxidant Components, but Through Distinct Mode of Actions

Applying UVA radiation has proven to be an innovative and effective tool to biofortify horticultural crops with enhanced nutraceuticals [5,10,13]. Nevertheless, there is currently a lack of information concerning plant responses to UVA irradiation patterns, underscoring the imperative of the current work to bridge this knowledge gap. Results from the second trial clearly illustrated that the irradiation patterns significantly regulated the growth and photochemical components of CCM in different ways: FI is efficient in boosting plants growth, being supported by larger plant morphology (Figure S3), high levels of biomass, *Chl a*, and *Chl a*/*b* ratio (Figure 3a,b,e), whereas SI favored enhancements of antioxidant components and TAC (Figure 4 and Figure 5).

**Figure 6:**
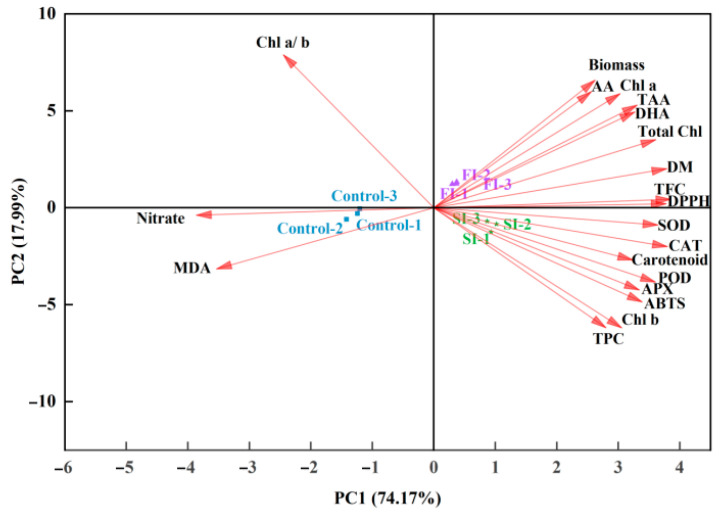
Principal component analysis (PCA) of related indicators under the influence of UVA-LED irradiation patterns. (DM—dry matter; *Chl a*—Chlorophyll a; *Chl b*—Chlorophyll b; *Chl a*/*b*—Chlorophyll a/*b*; Total *Chl*—total chlorophyll; TAA—total ascorbic acid; AA—ascorbic acid; DHA—dehydroascorbic acid; TPC—total phenolics content; TFC—total flavonoids content; ABTS-2,2′—azino-bis (3-ethylbenzothiazoline-6-sulfonic acid); DPPH-2,2-diphenyl-1-picrylhydrazyl; SOD—superoxide dismutase; POD—peroxidase; CAT—catalase; APX—ascorbate peroxidase; MDA—Malonaldehyde).

**Figure 7:**
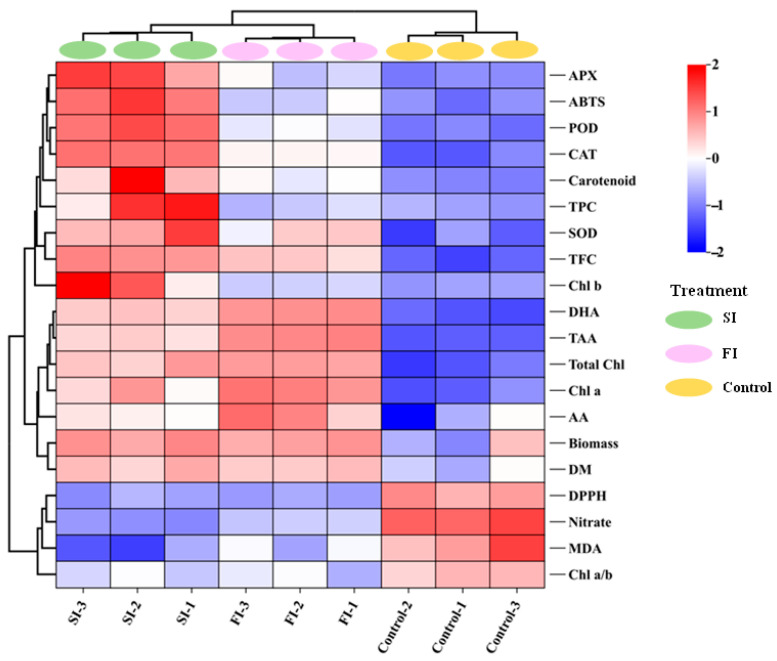
Heatmap analysis showing the comprehensive summary of different nutrients in CCM using a false color scale, with red indicating the highest (2), and blue the lowest value (−2) of each parameter. (DM—dry matter; *Chl a*—Chlorophyll a; *Chl b*—Chlorophyll b; *Chl a*/*b*—Chlorophyll a/*b*; Total *Chl*—total chlorophyll; AA—ascorbic acid; TAA—total ascorbic acid; DHA—dehydroascorbic acid; TPC—total phenolics content; TFC—total flavonoids content; ABTS-2,2′—azino-bis (3-ethylbenzothiazoline-6-sulfonic acid); DPPH-2,2-diphenyl-1-picrylhydrazyl; SOD—superoxide dismutase; POD—peroxidase; CAT—catalase; APX—ascorbate peroxidase; MDA—Malonaldehyde).

To further visualize the responses of CCM to UVA-LED radiation patterns, PCA was conducted followed by K-means clustering (Figure 6 and Figure 7; Table S3). As shown in Figure 6 and Table S3, the first two principal components (PC1 and PC2) exhibited eigenvalues exceeding 3 and collectively accounted for over 92% of the total variance. Specifically, PC1 and PC2 explained 74.17% and 17.99% of the variance, respectively, indicating that majority of the variability within the dataset could be effectively represented within a two-dimensional principal component space. The PCA scatter plot revealed three distinct treatment clusters, of which SI and FI treatments located closely together in the right quadrants and clearly separated from the control group, which positioned in the left quadrants (Figure 6). This spatial separation suggested that UVA-LED irradiation patterns had a notable impact on the overall growth and antioxidants profile of CCM, with each pattern influencing CCM in a distinct manner. Clearly, SI was associated with elevated levels of TPC, *Chl b*, ABTS, carotenoids, and antioxidant enzymes, whereas FI was profiled by biomass, AA, TAA, DHA, *Chl a*, total *Chl*, and DM. In contrast, the control group, which was clearly separated from both FI and SI, displayed high level of nitrate and MDA (Figure 6). In agreement to the PCA, hierarchical clustering analysis also demonstrated that the FI and SI treatments formed a tightly grouped cluster based on growth traits and antioxidant profiles (Figure 7). This close association indicates that both FI and SI patterns applied in this study elicited comparable physiological and biochemical changes in CCM plants. However, each irradiation pattern had distinct effects: the FI treatment was more favorable to growth traits, while the SI treatment was more effective in stimulating antioxidant properties. On the contrary, the control cluster was distinctly separated from the FI and SI clusters, reflecting its significant reduction in yield and nutrients (Figure 7). Overall, the clustering heatmap supported the PCA results, reinforcing the conclusion that UVA-LED radiation exerted a substantial influence on growth traits and antioxidant characteristics of CCM. Notably, this influence varies depending on the specific irradiation patterns, with FI characterized by multi-pulses irradiation at low dose appeared to promote CCM growth performance, whereas SI characterized by single pulse irradiation at high dose activated antioxidant system (Figure 3, Figure 4 and Figure 5).

Under low light condition, UVA can enhance photosynthesis, thereby increasing the photosynthetic carbon assimilation. However, high levels of UVA irradiation have been reported to be harmful for plant photosynthesis, targeting the photosystem II complex [9]. In our study, FI treatments, characterized by four pulses at low dose of 8 J/cm^2^ each (Figure 1d), enhanced the growth performance and agronomy traits. This enhancement could be achieved by the direct absorption of UVA by photosynthetic pigments, as the wavelength peak of UVA-LED applied in this study is at 390 nm (Figure 1b), which is very close to 400 nm, near that UVA has relatively higher light-use efficiency in terms of photosynthesis [29]. In fact, as the UVA wavelength approaches 400 nm, its promotive effect on the photosynthetic rate becomes more pronounced [10]. Additionally, UVA and blue light can stimulate stomatal aperture and chloroplast relocation, mediated by specific light receptors known as phototropins (Phot 1 and Phot 2) [31]. These responses probably increase CO_2_ availability and uptake, contributing to photosynthesis, and consequently accelerate plant growth performance observed under the FI treatment.

On the contrary, SI treatment, characterized by single pulse at high dose of 32 J/cm^2^ (Figure 1d), significantly stimulated the production of both enzymatic (SOD, CAT, POD, and APX) and non-enzymatic (TPC, TF, and carotenoids) antioxidants, along with an overall increase in TAC (Figure 4d–g, Figure 5a–d). This suggests that single pulse at high dose UVA-LED might trigger stress-defense responses; in turn, the plant activates defensive mechanisms by enhancing the levels of antioxidant components. This distinct plant response highlights the differing modes of action between the following two irradiation patterns: the FI treatment primarily promotes growth performance, whereas the SI enhances antioxidant property, despite both treatments finally delivering an equivalent total dose of 32 J/cm^2^. This provides an option for producers to target implementation in commercial production. FI approach is more appropriate for conventional production where maximizing output and economic return are the primary objectives. In contrast, SI strategy is particularly suitable for production of targeting high-value and functional foods. Consequently, the respective irradiation pattern can be selected based on a “quality-first” or “yield-first” strategy. Differing from our results, Silva et al. [17] found that a single UVC (254 nm) pulse of 0.3 kJ/m^2^ improved biomass and maintained a good physiological state of red mustard microgreens. However, three pulses of 0.3 kJ/m^2^ each negated this benefit, with plant responses reverting to control levels, suggesting potential inhibitory effect or even oxidative damage at higher cumulative doses. Interestingly, in our study, plants exposed to the SI treatment did not exhibit such a regression. On the contrary, they showed significantly enhanced antioxidant profiles and improved growth performance compared to the control. This discrepancy might be attributed to the difference in UV type applied. Unlike UVC, which has high energy and is often harmful to plant tissues at elevated doses, UVA possesses lower energy and is generally less damaging to plants.

### 4.3. UVA-LED Improves the Antioxidant Property of CCM via Accumulating the Antioxidants

**Figure 8:**
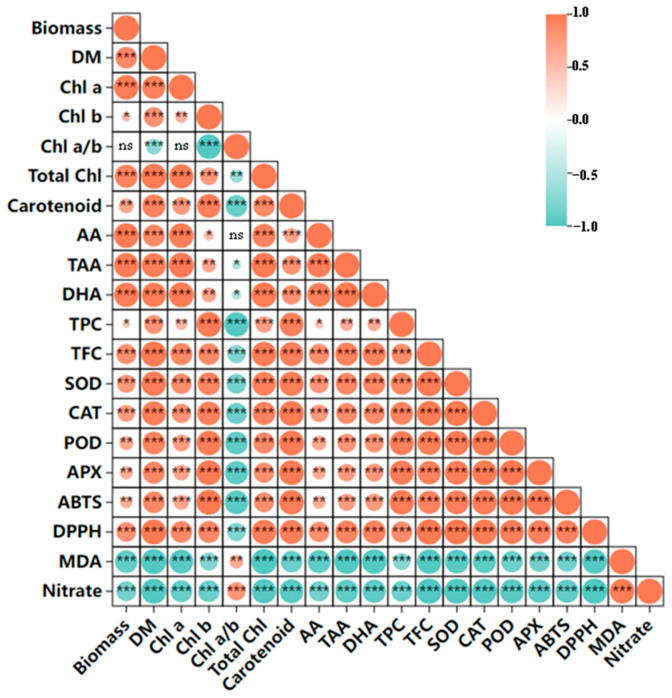
The Pearson’s correlations analysis among selected variables. The correlation coefficients are proportional to the color intensity. Positive correlation is displayed in red and negative in blue color. *, **, *** represent that the correlation is significant at the 0.05, 0.01, and 0.001 level (two-tailed). ns—not significant. (DM—dry matter; *Chl a*—Chlorophyll a; *Chl b*—Chlorophyll b; *Chl a*/*b*—Chlorophyll a/b; Total *Chl*—total chlorophyll; AA—ascorbic acid; TAA—total ascorbic acid; DHA—dehydroascorbic acid; TPC—total phenolics content; TFC—total flavonoids content; ABTS-2,2′—azino-bis (3-ethylbenzothiazoline-6-sulfonic acid); DPPH-2,2-diphenyl-1-picrylhydrazyl; SOD—superoxide dismutase; POD—peroxidase; CAT—catalase; APX—ascorbate peroxidase; MDA—Malonaldehyde).

In the present study, two widely used methods, ABTS and DPPH, were applied to assay the TAC of CCM, both based on free radical scavenging activity. As expected, TAC values obtained from the two methods showed a strong consistency (R = 0.94, *p* < 0.001) (Figure 8), indicating the reliability and feasibility of these methods for monitoring the antioxidant potential of CCM. UVA-LED irradiation, particularly under the SI pattern, significantly elevated TAC levels compared to the control (Figure 4g,h). This elevation was primarily attributed to the enhanced levels of both enzymatic (SOD, CAT, POD, and APX) and non-enzymatic antioxidants (TAA, AA, TP, TFC, and carotenoids), as all of them exhibited extremely positive correlations with TAC (Figure 8). These results are in line with previous findings by Brazaitytė et al. [5] who observed all supplemental UVA-LED increased the DPPH radical scavenging activity of microgreens, particularly at higher intensity level (12 µmol·m^−2^ s^−1^), by accumulating antioxidants-tocopherol level. Fan et al. [18] also demonstrated that the application of UVA-LED at a proper dose effectively stimulates both enzymatic and non-enzymatic antioxidant system in postharvest pakchoi by stimulating de novo synthesis of phenolic acids and flavonoids compounds, via activating the key enzymes and upregulation gene expression in phenylpropanoid pathway. However, a limitation of the present study is that specific phenolic acids and flavonoid compounds were not identified. This omission restricts an underlying mechanistic understanding of the pathways involved in polyphenolic compound accumulation under UVA-LED treatment, particularly under the SI pattern, which induced a marked increase in TPC and TFC (Figure 4d,e). Therefore, future studies are urgently needed to investigate the specific profiles and metabolic pathways of phenolic acids and flavonoids in response to different UVA-LED irradiation patterns.

It is worth noting that TAA, known as vitamin C, is a potent non-enzymatic antioxidant. It plays a critical role in scavenging reactive oxygen species (ROS), thereby mitigating oxidative damage from environmental stressors such as high UV irradiation. Exposure of plants to high levels of UV light can enhance the production of TAA as a protective response to mitigate UV-induced oxidative stress [32]. In our study, given that the SI treatment involved a single pulse at a higher dose, it would be reasonable to expect that SI would result in a higher level of TAA content. However, contrary to expectations, plants under the SI treatment exhibited a lower TAA content compared to those under FI treatment (Figure 4a). One possible explanation lies in the accumulation of high levels of secondary metabolites such as TPC and TFC in SI-treated plants (Figure 4d,e). According to the growth-defense trade-off hypothesis, plants with limited energy and resources must strategically allocate them between growth (biosynthesis of primary metabolites like TAA) and defense (secondary metabolites production, like TPC and TFC). Thus, the enhanced synthesis of secondary metabolites in SI-treated plants might have occurred at the expense of TAA accumulation. Additionally, in FI-treated plants, the high chlorophyll a, an essential photosynthetic pigment that efficiently absorbs blue-violet light, might improve the availability of soluble sugars, the initial precursor of AA biosynthesis, consequently leading to the increase in TAA level [33]. This speculation was further supported by the strong positive correlations observed between TAA and photosynthetic pigments (Figure 8). Similarly to our findings, Brazaitytė et al. [5] reported that high-intensity UVA-LED (366 nm, 12.4 μmol m^−2^ s^−1^) negatively affected ascorbic acid levels in basil and beet microgreens compared to lower intensities. He et al. [30] also found that high-dose UVA exposure (15 W/m^2^) reduced vitamin C content in Chinese kale relative to medium doses (10 W/m^2^). Nevertheless, the impact of UVA on vitamin C content remains inconsistent across the literature. For example, Zhu et al. [34] showed that supplemental UVA (368 nm, 60 µmol m^−2^ s^−1^) increased vitamin C levels by 30% in red lettuce cv. ‘Red Salad Bowl’, but had no significant effect on cv. ‘Rouxai’. Brazaitytė et al. [5] found that among three UVA-LED wavelengths tested (366, 390, and 402 nm), only 402 nm treatment significantly increased ascorbic acid content in mustard microgreens. These discrepancies highlight the complexity of UVA regulation of vitamin C and present a challenge to draw a conclusion. Despite this, it is clear that preharvest UVA application significantly influences the dynamics of vitamin C content in leafy greens. This effect results from the interplay of multiple regulatory factors, including plant species or even cultivar, UVA peak wavelength, irradiance intensity, and irradiation pattern.

### 4.4. The Nitrate Content in CCM Is Far Below the Threshold of Trade Limitation and Reduced by UVA-LED Regardless of Irradiation Patterns

Nitrate content is a critical quality index of vegetables, with elevated levels that may pose potential health risks to consumers. Numerous factors affect nitrate uptake and accumulation in vegetables, including genetic background, environmental conditions, and agricultural practices. Among these, nitrogen fertilization and light irradiation have been identified as the primary determinants of nitrate accumulation [35,36]. In Europe, the maximum nitrate concentration permitted in vegetables ranges from 1500 to 5000 mg/kg fresh weight, depending on the species [36]. In China, the recommended safety limit for leafy vegetables is 3000 mg/kg FW [37]. Our result showed that the highest nitrate content observed in the control sample was 347.77 mg/kg FW (Figure 5f), which is far below the threshold of 1500 mg/kg fresh weight trade limitation for cabbage [36]. This result suggested that all CCMs are safe for consumption and do not pose a health risk related to nitrate toxicity. Such a low nitrate level observed in CCM was possibly attributed to the absence of nitrogen fertilization during CCM cultivation. As described in Section 2.1.1, no fertilizer, but only tap water, was applied during CCM growth. This assumption was in agreement with the findings of Liu et al. [38] who reported that preharvest treatments with nitrogen-free solutions (distilled water) effectively reduced nitrate concentration in lettuce. Therefore, for short-cycle microgreens production, the use of tap water in place of nutrient solution could present a practical and cost-effective alternative to lowering nitrate accumulation.

Additionally, light irradiation is another determinant influencing nitrate intake, assimilation, and distribution in plant [35]. However, the effect of UVA light on nitrate content in leafy vegetables remains controversial and poorly understood. Brazaitytė et al. [11] reported UV-A at 366 nm and 390 nm led to an increase in nitrate content, whereas 420 nm UVA maintained constant nitrate level in mustard microgreens. He et al. [30] found low dose (380 nm, 5 W/m^2^) UVA increased nitrate levels in kale, while medium (10 W/m^2^) and high doses (15 W/m^2^) showed no significant effects. In our study, the nitrate content in CCM was significantly reduced by UVA-LED treatment regardless of irradiation patterns (Figure 5f). Similarly to our observations, the nitrate reduction in Chinese kale following UVA exposure was observed by Hu et al. [39]. This reduction might be attributed to UVA-induced upregulation of nitrate reductase (NR) activity, a key enzyme involved in nitrogen metabolism [39]. As it is known, plants possess distinct signaling pathways in regulating nitrate accumulation by activating specific photoreceptors [35]. Cryptochromes (CRY) and phototropins (PHOT) are the best-known photoreceptors for UVA/blue light perception [40]. Upon activation by UVA/blue light, CRY proteins are phosphorylated form homodimers and undergo a conformational change, allowing physical interactions with signaling factors such as COP1. This interaction promotes downstream signal cascades involved in nitrate assimilation [35,40]. Despite this mechanistic framework, the precise molecular pathways through which UVA/blue light regulates nitrate metabolism remain largely unrevealed [35].

### 4.5. Research Limitations

The present study demonstrated that UVA-LED effectively regulates the growth and quality of CCM. Nevertheless, several limitations should be acknowledged. First, there is a notable technical constraint in the experimental setup. Due to the commercial unavailability of integrated UVA-LED and white light systems, UVA-LED and background white light source were physically separated in this study. Although this design allowed isolation of the sole-impact of UVA-LED, it does not reflect natural growth conditions where plants receive full-spectrum solar radiation. Consequently, potential synergistic or antagonistic interactions between UVA-LED and background light remain unknown, which affects the accurate assessment of UVA-LED practical application. Second, such physical separation of light sources required moving plants between different light regimes during UVA-LED treatment. This handling, particularly under FI pattern, may not only introduce mechanical stress that could confound the plant responses to UVA-LED observed, but also renders this approach labor-intensive and economically unfeasible for scalable industrial production. Finally, this research primarily focused on physiological and quality responses of CCM to UVA-LED exposure. The absence of molecular investigations, particularly regarding gene expression patterns and the role of specific UVA/blue light photoreceptors, limits the understanding of the underlying mechanisms. Elucidating the transcriptional regulatory and light signaling pathways is essential for a deeper insight into how UVA-LED precisely modulates the observed phenotypic and quality attributes of CCM.

## 5. Conclusions

This study clearly illustrated that preharvest application of UVA-LED at doses ranging from 16 to 48 J/cm^2^ positively affected the growth performance and enhanced antioxidant quality of CCM. However, the effects were highly dose-dependent, with 32 J/cm^2^ identified as the most effective dose. Both FI and SI effectively promoted growth and enhanced antioxidant property via activation of antioxidant enzymes activities and accumulation of non-enzymatic antioxidant. Interesting, the two irradiation patterns exhibited distinct modes of action; FI was more effective in promoting growth-related traits, whereas SI more strongly stimulated the accumulation of antioxidant-related components. This study makes notable contributions to the existing literature by emphasizing the importance of irradiation patterns to align with targeted production goals in the practical application of UVA-LED technology in commercial indoor agriculture and the food industry.

It is also worth emphasizing that production is only the first step in the fresh produce supply chain. Postharvest processing, storage, distribution, and marketing are equally critical for maintaining microgreens quality and safety. Consequently, future research should also explore the potential benefits of postharvest UVA-LED application to preserve or enhance microgreens quality throughout the entire supply chain—from farm to table.

## Data Availability

The original contributions presented in the study are included in the article/Supplementary Materials. Further inquiries can be directed to the corresponding authors.

## Supplementary Materials

The following supporting information can be downloaded at https://www.mdpi.com/article/10.3390/foods14234092/s1, Figure S1: Experiment 1, UVA-LED doses selection; Figure S2: Effect of UVA-LED irradiation doses on plant growth performance; Figure S3: Effect of UVA-LED irradiation pattern on growth performance; Table S1: Irradiation treatment of experiment 1; Table S2: Irradiation treatment of experiment 2; Table S3: Eigenvalues, factor scores, and contribution rates of the first two PCs to CCM variation under UVA-LED treatment.
