# Supplementary material for: Preharvest UVA-LED Enhancing Growth and Antioxidant Properties of Chinese Cabbage Microgreens: A Comparative Study of Single Versus Fractionated Irradiation Patterns
Source: Foods. 2025 Nov 28;14(23):4092. doi: 10.3390/foods14234092 (PMC12691816; doi:10.3390/foods14234092)
Supplement: Supplementary file 1 [file foods-14-04092-s001.zip › foods-3971596-supplementary.pdf]

Figure legends

Figure S1 Experiment 1, UVA-LED doses selection. DAS, days after sowing.

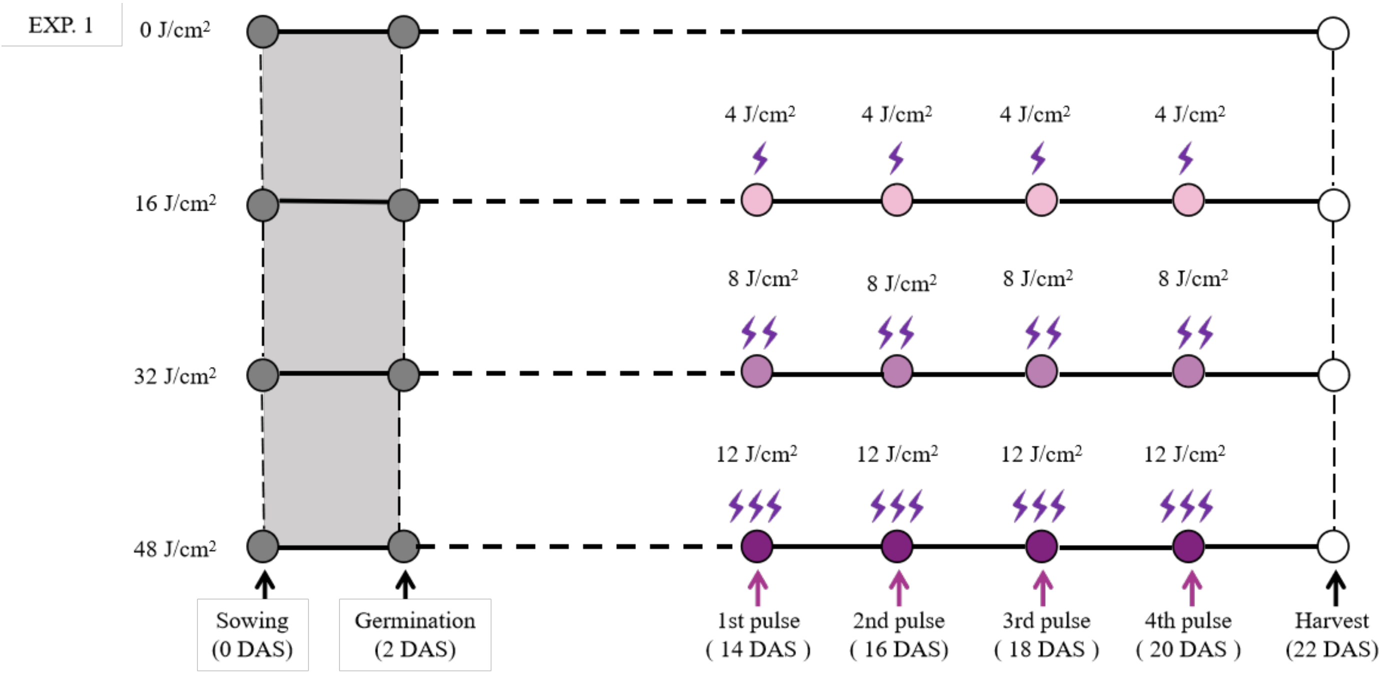

Figure S2 Effect of UVA-LED irradiation doses on plant growth performance.

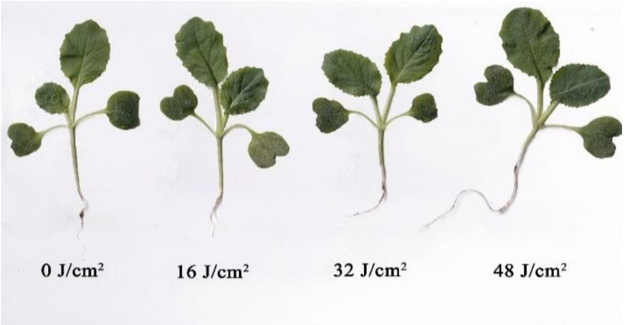

(a) Microgreens under treatments

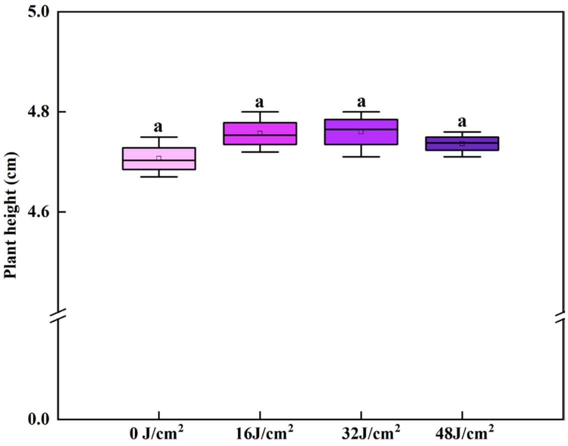

(b) Plants height

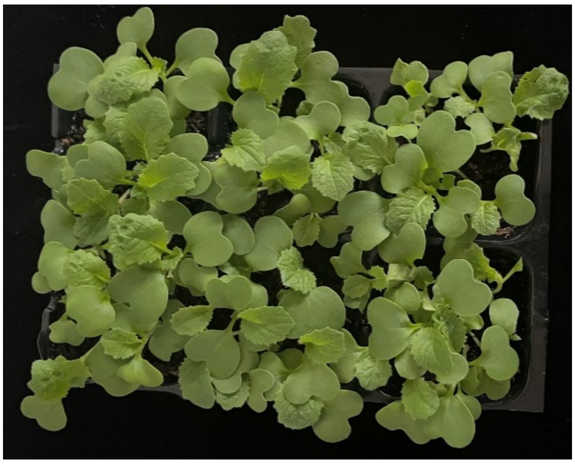

(c) 0 J/cm^2 (Control)

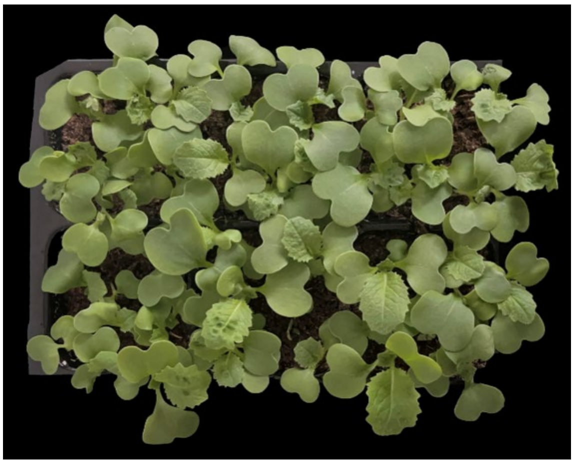

(d) 16 J/cm^2

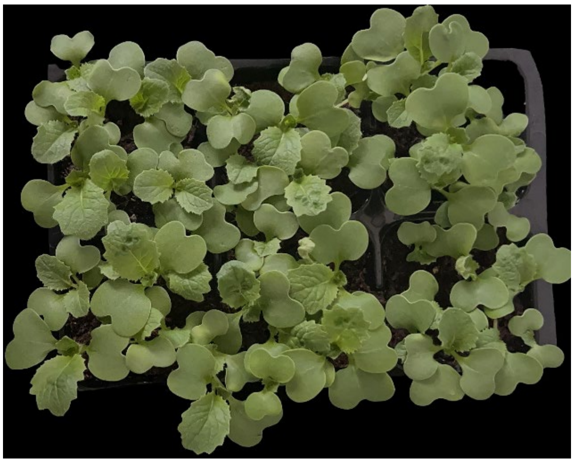

(e) 32 J/cm^2

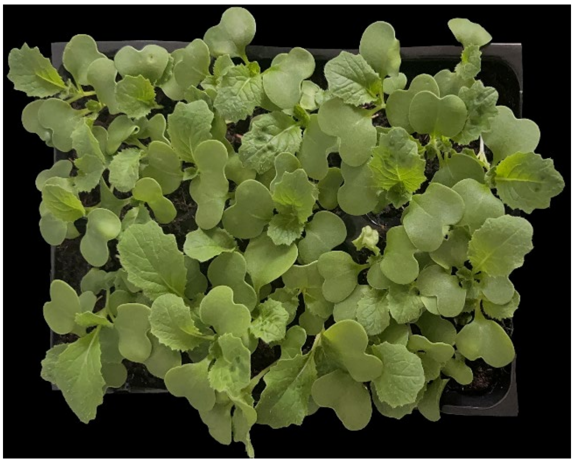

(f) 48 J/cm^2

Figure S3 Effect of UVA-LED irradiation pattern on growth performance. FI, fractionated irradiation; SI, single irradiation.

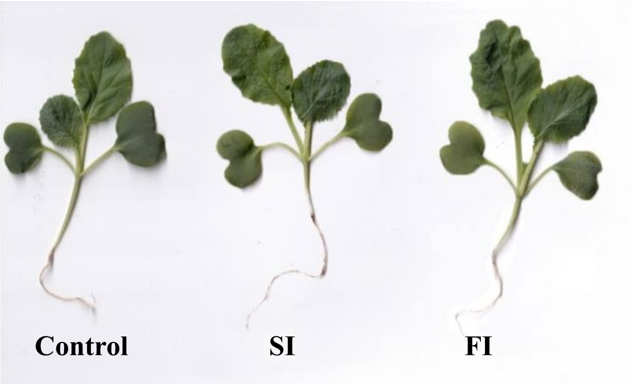

(a) Microgreens under treatments

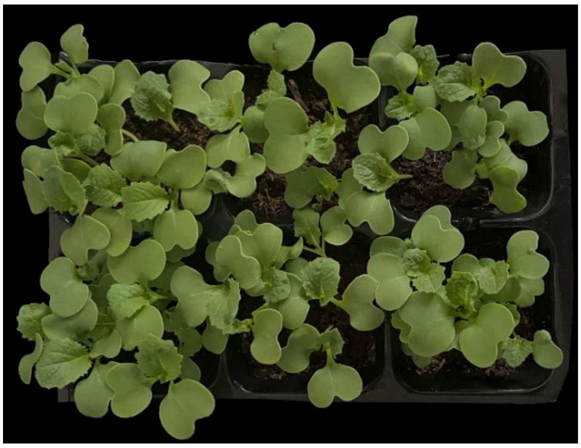

(b) Control

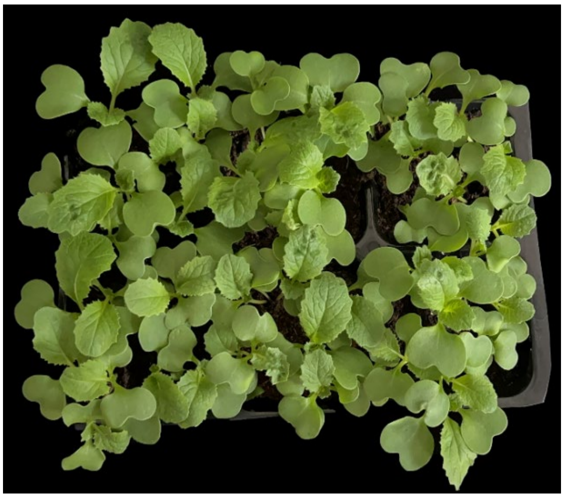

(c) SI

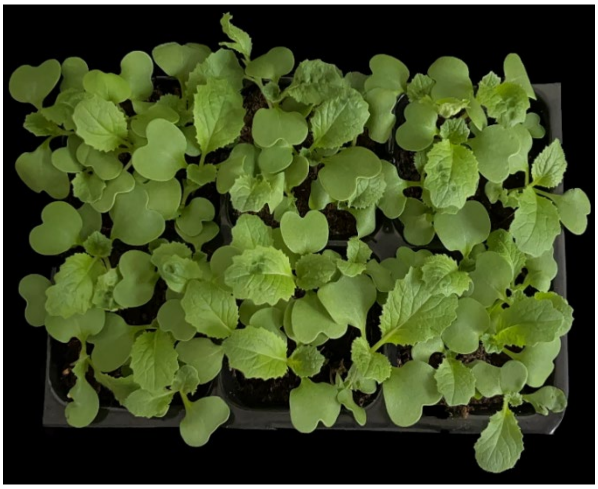

(d) FI

Table S1 Irradiation treatment of experiment 1

Dose (J/cm²)	Intensity (mW/cm²)	Each irradiation duration (minutes)	Total irradiation duration (minutes)
0 (Control)	0	0	0
16 (4*4)	12	5.56 (5 min and 34 s)	22.24
32 (4*8)	12	11.11 (11 min and 7 s)	44.44
48 (4*12)	12	16.67 (16 min and 40 s)	66.68

Table S2 Irradiation treatment of experiment 2.

Dose (J/cm²)	Intensity (mW/cm²)	Each irradiation duration (minutes)	Total irradiation duration (minutes)
0 (Control)	0	0	0
SI (32)	12	44.44 (44 min and 26 s)	44.44
FI (4*8)	12	11.11 (11 min and 7 s)	44.44

FI, fractionated irradiation; SI, single irradiation.

Table S3 Eigenvalues, factor scores, and contribution rates of the first two PCs to CCM variation under UVA-LED treatment.

Parameter	PC1	PC2
Biomass	0.17481	0.32906
DM	0.25257	0.10073
Chl a	0.20189	0.29431
Chl b	0.20361	-0.30945
Chl a/b	-0.16306	0.39427
Total Chl	0.24058	0.17554
Carotenoid	0.21568	-0.13464
AA	0.17072	0.2994
TAA	0.22027	0.26479
DHA	0.21781	0.24712
TPC	0.18656	-0.30932
TFC	0.25692	0.02194
SOD	0.2257	-0.24268
CAT	0.24285	-0.04426
POD	0.2532	-0.10014
APX	0.24025	-0.19143
ABTS	0.25187	0.01011
DPPH	0.2227	-0.212
MDA	-0.23558	-0.15775
Nitrate	-0.25739	-0.01903
Eigenvalue	14.83402	3.59723
Percentage of Variance (%)	74.17011	17.98616
Cumulative (%)	74.17011	92.15628
